# Empowering High-Throughput High-Content Analysis of Microphysiological Models: Open-Source Software for Automated Image Analysis of Microvessel Formation and Cell Invasion

**DOI:** 10.1007/s12195-024-00821-2

**Published:** 2024-10-10

**Authors:** Noah Wiggin, Carson Cook, Mitchell Black, Ines Cadena, Salam Rahal-Arabi, Chandler L. Asnes, Yoanna Ivanova, Marian H Hettiaratchi, Laurel E Hind, Kaitlin C Fogg

**Affiliations:** 1 School of Electrical Engineering and Computer Science, Oregon State University, Corvallis, OR USA; 2 School of Chemical, Biological, and Environmental Engineering, Oregon State University, Corvallis, OR 97330 USA; 3 Phil and Penny Knight Campus for Accelerating Scientific Impact, University of Oregon, Eugene, OR USA; 4 Department of Chemistry and Biochemistry, University of Oregon, Eugene, OR USA; 5 Department of Chemical and Biological Engineering, University of Colorado Boulder, Boulder, CO USA

**Keywords:** Bioinformatics, Computational biology, Tumor microenvironment, Pharmacokinetics, Tissue engineering, Hydrogel

## Abstract

**Purpose:**

The primary aim of this study was to develop an open-source Python-based software for the automated analysis of dynamic cell behaviors in microphysiological models using non-confocal microscopy. This research seeks to address the existing gap in accessible tools for high-throughput analysis of endothelial tube formation and cell invasion in vitro, facilitating the rapid assessment of drug sensitivity.

**Methods:**

Our approach involved annotating over 1000 2 mm Z-stacks of cancer and endothelial cell co-culture model and training machine learning models to automatically calculate cell coverage, cancer invasion depth, and microvessel dynamics. Specifically, cell coverage area was computed using focus stacking and Gaussian mixture models to generate thresholded Z-projections. Cancer invasion depth was determined using a ResNet-50 binary classification model, identifying which Z-planes contained invaded cells and measuring the total invasion depth. Lastly, microvessel dynamics were assessed through a U-Net Xception-style segmentation model for vessel prediction, the DisPerSE algorithm to extract an embedded graph, then graph analysis to quantify microvessel length and connectivity. To further validate our software, we reanalyzed an image set from a high-throughput drug screen involving a chemotherapy agent on a 3D cervical and endothelial co-culture model. Lastly, we applied this software to two naive image datasets from coculture lumen and microvascular fragment models.

**Results:**

The software accurately measured cell coverage, cancer invasion, and microvessel length, yielding drug sensitivity IC_50_ values with a 95% confidence level compared to manual calculations. This approach significantly reduced the image processing time from weeks down to h. Furthermore, the software was able to calculate cell coverage, microvessel length, and invasion depth from two additional microphysiological models that were imaged with confocal microscopy, highlighting the versatility of the software.

**Conclusions:**

Our free and open source software offers an automated solution for quantifying 3D cell behavior in microphysiological models assessed using non-confocal microscopy, providing the broader Cellular and Molecular Bioengineering community with an alternative to standard confocal microscopy paired with proprietary software.This software can be found in our GitHub repository: https://github.com/fogg-lab/tissue-model-analysis-tools.

**Supplementary Information:**

The online version contains supplementary material available at 10.1007/s12195-024-00821-2.

## Introduction

Cancer is a complex disease characterized by uncontrolled cell growth, invasion, and metastasis [1]. Understanding the mechanisms underlying these processes is critical for developing effective treatment strategies. 3D tumor models are a useful tool to capture the interactions between tumor cells and the surrounding microenvironment, which play a key role in tumor progression [2]. However, there remains a lack of open-source image analysis software for automated assessment of dynamic tumor behaviors like growth, invasion, and angiogenesis [3]. This is a bottleneck for high throughput imaging experiments, hindering our ability to fully exploit the potential of 3D models to study these cell behaviors under various experimental conditions.

Common bioimage analysis software tools such as Fiji ImageJ [4] provide a diverse array of tools and a graphical user interface for detailed analysis of images. However, the manual work required to use these tools may increase in proportion to the number of images in the dataset, limiting their application for high throughput image analysis. Recent advancements in machine learning (ML) for computer vision have created new pathways for automated and accurate analysis of biological images. Deep learning has shown exceptional promise in image recognition and segmentation tasks in the domain of biomedical image analysis. These techniques have been used successfully in various applications, such as in the classification of invasion depth in esophageal squamous cell carcinoma [5] and in cell coverage area analysis in time-lapse fluorescence microscopy [6].

Deep learning models such as U-Net [7, 8] have demonstrated high accuracy in vessel segmentation tasks. Further accuracy may be obtained by considering the structural characteristics of a vessel network, such as graphical connectivity [9] Additionally, analyzing the structural properties of microvascular networks reveals phenotypic information of significant biological relevance [10] An emerging area in the quantitative analysis of biological networks is the application of topological data analysis techniques [11, 12]. In biological imaging, topological data analysis tools such as persistent homology have been used to characterize and quantify complex structures, such as the branching architecture of microvessel networks in brain artery trees [13] and neuronal structures [14]. In recent years, topological data analysis has been applied to characterize the structural and functional characteristics of vessel networks, including tumor vascular networks [15, 16].

Despite these advancements, there remains a need for comprehensive, open-source tools designed explicitly for measuring cell behaviors of 3D models in high throughput non-confocal imaging experiments. The development of such tools would facilitate studies into cell-microenvironment interactions in 3D cultures and contribute towards understanding the effect of the tumor microenvironment on cancer progression.

Here we introduce an open-source software application for the high-throughput analysis of cancer and endothelial cell dynamics in hydrogels. We applied this software to 3D multilayer multicellular models of cervical and endometrial cancer cells co-cultured with human microvascular endothelial cells (hMVEC). Our software application automates the quantification of cell coverage area, invasion depth, and microvessel formation, enabling rapid and accurate assessment of phenotypic cell responses in 3D tumor models.

## Materials and Methods

### Cell Lines and Reagents

Unless stated, all reagents were purchased from ThermoFisher (Waltham, MA). Human microvascular endothelial cells (hMVEC) were purchased from Lonza (hMVEC 33226, Walkersville, MD) and used without additional characterization. Cells were expanded in EGM-2 MV media (EBM-2 supplemented with Lonza's SingleQuot supplements: hydrocortisone, human basic fibroblast growth factor (FGF2), human vascular endothelial growth factor (VEGF), human insulin-like growth factor (IGF), human epidermal growth factor (EGF), ascorbic acid, and gentamycin) and further supplemented with 5% fetal bovine serum (FBS) until used at passage 5. Human cervical cancer cell lines SiHa (ATCC® HTB-35™) and Ca Ski (ATCC CRM-CRL-1550) and human endometrial cancer cell line HEC-1A (ATCC HTB-112™) were purchased from ATCC (Manassas, VA) and used without additional characterization. The cells were cultured in 1% penicillin–streptomycin (Sigma-Aldrich, St. Louis, MO, USA) and 10% fetal bovine serum maintaining Eagle's Minimum Essential Medium (EMEM, ATCC), RPMI-1640 Medium (ATCC), and McCoy’s 5A medium (ATCC) respectively until used at passage 5. All cell types were expanded in standard cell culture conditions (37 °C, 21% O_2_, 5% CO_2_) and subcultured before they reached 80% confluency.

### Multilayer Hydrogel Fabrication

Multilayer multicellular constructs for cervical cancer cells and endometrial cancer cells were prepared following the same methodology as previously reported (Table 1) [17].

**Table 1:** Summary of hydrogel formulations for cervical and endometrial cancer models

Physiomimetic model	Bottom hydrogel composition	Top hydrogel composition
Cervical cancer	7% w/v GelMA, 2.5 mg/mL fibrinogen, 2.5 mg/mL Col1	7% w/v GelMA, 1.12 mg/mL Col1, 0.16 mg/mL fibronectin
Endometrial cancer	8.7% w/v GelMa, 0.6 mg/mL fibrinogen, 1.9 mg/mL Col1	10% w/v PEGDA, 0.13 mg/mL Col IV, 0.17 mg/mL fibronectin, 0.5 µg/mL laminin

Constructs were fabricated in specialized µ-Plate Angiogenesis 96-wells, ibidi Treat (ibidi, Munich, Germany). Before seeding, endothelial cells were labeled green using 1 μM CellTracker™ Green CMFDA Dye and cancer cells were labeled red using 1 μM CellTracker™ Red CMTPX Dye according to manufacturer’s protocols and without further characterization of the dyes. The multilayer multicellular construct was formed by layering 10 µL of the bottom hydrogel formulation into each well, and 20,000 CellTracker Green labeled hMVEC cells in 40 µL of EGM-2 MV were pipetted on top of each gel. The endothelial cells were allowed to attach for four h at 37 °C and 5% CO_2_. The media was then carefully removed, and 25 µL of the top hydrogel formulation was pipetted on top. Finally, cell tracker Red-labeled cancer cells were seeded on top of each gel at 10,000 cells in 25 µL of media. As a control, multilayer Matrigel constructs were made using the same methods described above, except the custom hydrogel formulations were replaced with growth factor deficient Matrigel basement membrane matrix (9.2 mg/mL protein concentration, Corning, MA, USA) in both layers. This resulted in the cancer cells being seeded approximately 1750 μm above the bottom of the IBIDI wells. The media was carefully changed every 12 h, and the plates were incubated at 37 °C and 5% CO_2_ in a BioSpa live cell analysis system (Agilent Technologies, Santa Clara, CA) for 45 h.

### Phenotypic Cell Response to Paclitaxel

Using the optimized cervical cancer construct [17], we measured the phenotypic cell responses: microvessel length, cervical cancer invasion, endothelial cell coverage, and cancer cell coverage in endothelial cells (hMVEC), and the cervical cancer cells (SiHa and Ca Ski) cultured the multilayer multicellular models and treated with the chemotherapy agent Paclitaxel for 48 h. Paclitaxel was a kind gift from the Oregon State University College of Pharmacy High-Throughput Screening Services Laboratory. Cells were imaged every 12 h, and the cell response to the drug was reported at 24 h of treatment. The dose-response analysis of Paclitaxel ranged from 0.008 to 25 μM, and dimethyl sulfoxide (DMSO) was used as a vehicle. Paclitaxel and DMSO were dispensed in the wells using an automated liquid handler (D300e Digital Dispenser, Hewlett Packard, Corvallis, OR). The dose response-inhibition curves were calculated using Prism 8.2.1 software (GraphPad, San Diego, CA). We then evaluated the IC_50_ values in terms of phenotypic responses. While IC_50_ values are classically defined as the concentration that inhibits 50% of the cells from proliferating or the concentration that kills 50% of the cells, here we defined the IC_50_ values as the concentration of drug that reduced either cancer invasion, cancer coverage, microvessel length, or endothelial cell coverage to 50% of the value observed in the absence of drug. Lastly, we compared previously reported IC_50_ values using Fiji ImageJ (NIH, Bethesda, MD) and the Gen5 software (Agilent Technologies) [17] with the described image analysis tools.

### Image Acquisition in Multilayer Multicellular Model

We have previously demonstrated that our multilayer multicellular 3D in vitro model can support the phenotypic cell response over time [17]. The phenotypic responses were defined as cancer cell coverage area, cancer cell invasion, endothelial cell coverage area, and endothelial microvessel length. Samples were imaged at the Nyquist optimized step size, and two-channel 85 µm z-stack images were taken using a 4X Universal Plan Fluorite Phase objective, Numerical Aperture 0.13, Working Distance 17 mm with a Cytation 5 V3.14 cell imaging multi-mode reader (Agilent Technologies). A total of 21 z-stacks per well and per experiment were recorded and used for post-processing to calculate the cell phenotypic response in each hydrogel model.

### Software Application for Automated Image Analysis

We developed a Python software application that contains four automated image analysis tools designed to facilitate the quantification of phenotypic cell response in a high-throughput setting. Our application contains automated image analysis tools for assessing microvessel formation, cell coverage, and cancer invasion depth. This open-source software can be used as a standalone application that comes with an easy to use graphical user interface (GUI, Figure S1), as a command-line utility, or as an integrated component within other software programs.

Comprehensive documentation detailing setup and usage instructions, including options for customization, can be found in our GitHub repository:

### https://github.com/fogg-lab/tissue-model-analysis-tools.

We have provided an interactive demonstration of each analysis tool in the supplementary materials, which can be accessed via the Colab notebook link: https://colab.research.google.com/github/fogg-lab/tissue-model-analysis-tools/blob/main/notebooks/analysis_demo.ipynb

### Making Z-Projection Images

For manual calculations, the Cytation 5 a Z-projection function was used to combine the 21 Z-stacks into a 2D Z-projection. Using a focus stacking with a maximum filter size of 11 px, we captured the cell response in one 2D image. For automated calculations, we employed five established projection methods to compute a Z-projection of input Z-stacks. These methods include focus stacking, minimum pixel intensity, maximum pixel intensity, median pixel intensity, and average pixel intensity. Users can select the most suitable method for computing Z-projections of their samples, depending on the characteristics of their dataset. To compare the phenotypic cell response between manual inspection and automated analysis, we chose the focus stacking approach, generating an output comparable to that of Gen5.

### Manual Inspection of Cell Coverage Area

After computing the Z-projections with Gen5, images were processed with NIH Fiji-ImageJ (NIH, Bethesda, MD). Cell coverage was measured for each cell type at every time point by calculating the area within the well covered by cells and dividing it by the total well area. A macro script was generated to process a batch of images over time (Figure S1). Cell coverage was reported as a percentage of the endothelial cells in the well as well as the percentage of cancer cells in the well.

### Automated Computation of Cell Coverage Area

We implemented a procedure in the software that incorporates a two-component Gaussian Mixture Model (GMM). Two Gaussian curves are fit to the pixel intensities in the image, with one curve fit to the foreground pixels and the other fit the background pixels. The foreground pixels are defined as the bright regions in the image (high pixel intensities), indicating cells, while the background comprises the darker regions without cells (low pixel intensities).

**Fig. 1:**
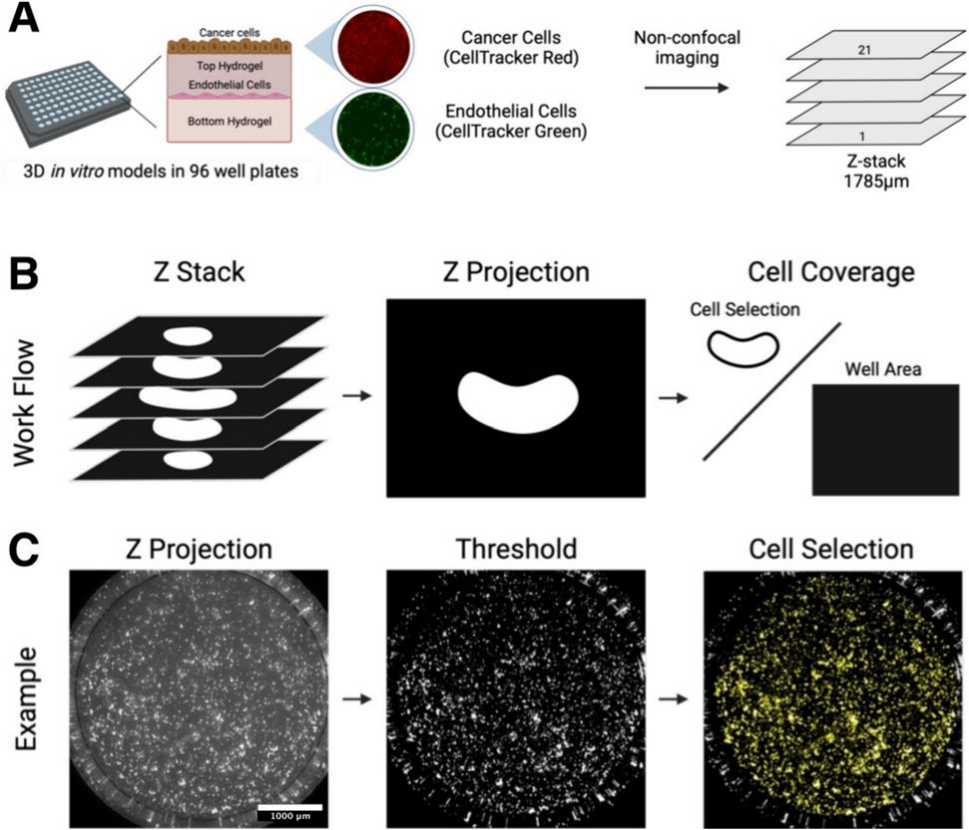
Schematic representation of image acquisition and cell thresholding.** A** CellTracker Green labeled human microvascular endothelial cells (hMVEC) were cocultured in different 3D *in vitro* hydrogel models with CellTracker Red labeled cancer cells (SiHa, CaSki, or HEC-1A). Constructs were imaged every three h for 48 h with two different channels (GFP and Texas Red) and a 1785 µm Z-stack was taken for each well to capture full construct height. **B** Schematic demonstrating Z-projection followed by cell coverage calculation **C** Example of how Z-projection images are used to calculate cell coverage.

After fitting the GMM, we then compute cell area by thresholding the image based on a cutoff pixel intensity calculated as ${\mu }_{foreground}+\lambda \times {\sigma }_{foreground}$. Here, ${\mu }_{foreground}$ and ${\sigma }_{foreground}$ represent the estimated mean and standard deviation of the foreground intensity, which are parameters extracted from the GMM’s foreground component. $\lambda$ is a tunable parameter used as a multiplier of the foreground standard deviation. When $\lambda =0$, pixels with intensities greater than ${\mu }_{foreground}$ pass the threshold. When $\lambda >0$, fewer pixels pass the threshold. When $\lambda <0$, more pixels pass the threshold. This approach is effective for images containing a bimodal distribution of pixel intensities, regardless of the overall brightness and contrast of each image. Similar to manual inspection, cell coverage was then calculated as the cell area divided by the total area of the well. (Fig. 1). The well boundary was determined by estimating the parameters of a superellipse that fits around the Canny edges of the image. If this failed due to background noise near the image border, or due to a lack of detected Canny edges, we used a circular boundary centered on the image.

### Manual Inspection of Cell Invasion Depth

Cell invasion depth was defined as the deepest point of cancer cell invasion after 24 h, and 48 h of cultured in the 3D multilayer model. This was achieved by annotating each Z-stack image set and calculating the difference between cell invasion from time 0 h, 24 h and 48 h. Using Gen5, we manually inspected each well and z-stack to track cell movement over time.

### Automated Computation of Cell Invasion depth

**Fig. 2:**
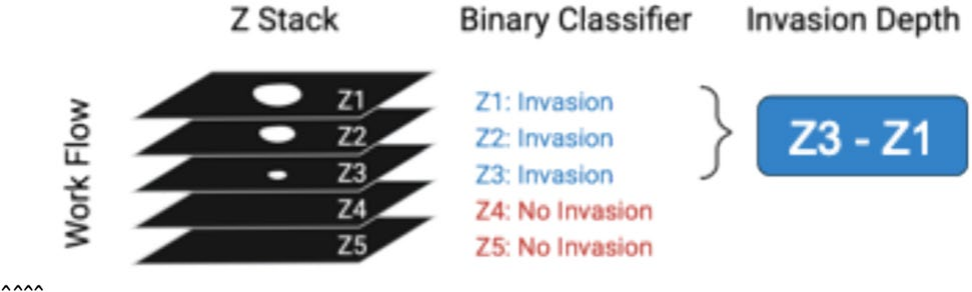
Schematic representation of invasion probability determination. For each input image, a binary classifier calculates the probability of invasion, with a probability greater than or equal to 0.5 indicating that the sample is classified as "Invaded." Invasion depth is then calculated as the distance between the top z plane and the deepest Z-plane with invasion.

We use a binary classifier deep neural network based on the ResNet50 architecture to estimate the depth of invasion in a given Z-stack. We trained the classifier on a dataset of 997 images, split into two classes - *invasion* and *no invasion*. 848 of these images were invaded, and 149 were not invaded (Fig. 2). During training, we augmented the dataset by applying random flips and rotations to the samples. The binary classifier assesses each image in a Z-stack to determine whether it displays a sufficient amount of in-focus cell area to be considered as demonstrating invasion. A Z-stack of input images $Z=({z}_{k},{z}_{k-1},...,{z}_{0})$ is processed by the invasion depth analysis system, which outputs two results:

1. A collection of probabilities, $\widehat{p}$, showing the model’s confidence that invasion has been identified:
  $$\widehat{p}=({p}_{k},{p}_{k-1},...,{p}_{0})$$
2. A collection of classifications, ${\widehat{y}}_{p}$, thresholded at a given value (typically ${p}_{i}>0.5$):
  $$\widehat{y}=({\widehat{y}}_{k},{\widehat{y}}_{k-1},...,{\widehat{y}}_{0})$$

### Manual Quantification of Microvessel Formation

Endothelial microvessel length was quantified by measuring the average length of the microvessels found in a well at each time point using NIH Fiji-ImageJ. We use a macro script to determine the number of microvessels, total branch length and average length of each branch in each set of Z-projected images (Supplemental Fig 2). The average branch length was reported in microns.

### Automated Quantification of Microvessel Formation

**Fig. 3:**
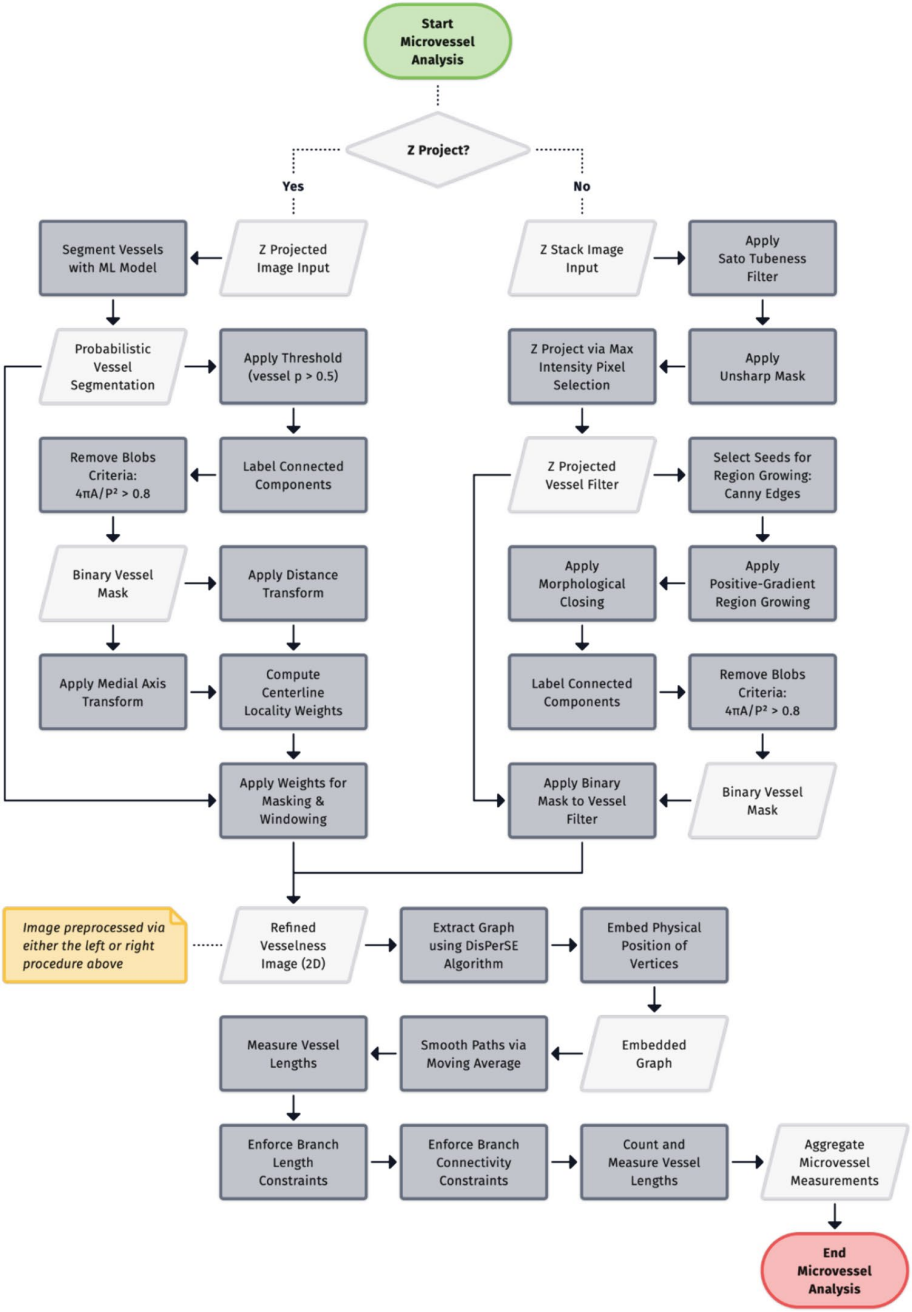
Workflow of the microvessel analysis pipeline. Input Z projections are segmented using a machine learning model, while input Z-stacks are filtered using a Sato tubeness filter. Either pathway produces a refined vesselness image, from which a graph representation is extracted using the DisPerSE algorithm. The resulting graph is then analyzed to measure vessel lengths. Branch length constraints are then applied and disconnected branches are then optionally removed. The pipeline outputs aggregate measurements of the microvessel network, including branch counts, average branch length, and total network length.

Endothelial microvessel length was quantified by preprocessing an image to enhance vessel centerlines, constructing a 2D geometric graph representation of the vessel network, and analyzing the embedded graph (Fig. 3). This process begins with input image preprocessing, which differs for Z-projections and Z-stack images. Z-projections are processed using a binary semantic segmentation model (section "Binary Semantic Segmentation") to generate a vessel probability map, while Z-stack images undergo Sato tubeness filtering [18] to enhance tubular structures.

Following initial preprocessing, the images undergo further processing steps to reduce noise and enhance vessel centerlines. Vessel probability maps (computed for Z-projections) and Sato-filtered Z-stacks require separate post-processing procedures to refine the microvessels as detailed in sections "Post-Processing Raw Segmentations Prior to Graph Extraction" and "Post-Processing Sato filter Z-Stack Prior to Graph Extraction". The DisPerSE algorithm [19] then extracts a graph representation, known as the Morse skeleton, of the microvessel network (section "Graph Extraction"). This extracted graph is analyzed to measure vessel lengths, branch counts, and other network characteristics, as described in section "Topological Data Analysis". The final output of this process consists of aggregate measurements of the microvessel network, including branch counts, average branch length, and total network length.

### Binary Semantic Segmentation

We trained a U-Net Xception-style model [8, 20] to segment the microvessels from the background of an image. The target output from this process is a probabilistic (non-thresholded) segmentation of the image, where each predicted value is the probability that the pixel at that position is part of a vessel. To train, validate, and test the segmentation model, we prepared a dataset of fifty manually annotated images. Each annotated image in the dataset consisted of the original image and its corresponding annotation in the form of a binary segmentation mask. We produced annotations for fifty images using an interactive segmentation program [21] based on the RITM: Interactive Segmentation codebase [22]. We split the annotated images into three datasets - a training set of thirty samples, a validation set of ten samples, and a test set of ten samples.

At training time, we applied a series of image transformations to each training sample as a form of data augmentation. These transformations improved the model’s convergence and were necessary for the model to generalize to images outside the training dataset. The transformations and the probabilities for applying each transformation were chosen experimentally. We applied the following image transformations to the training samples: rotate at a random angle ($p=0.5$), crop to a random 512×512 patch ($p=1.0$), flip horizontally ($p=0.25$) or vertically $(p=0.25$), alter brightness and contrast ($p=0.7$), apply either multiplicative noise ($p=0.4$) or gaussian blur with noise [23] $(p=0.4)$, and apply elastic deformations [24] ($p=0.85$). The transformed samples were subsequently resampled to the target size of 320×320. Lastly, each sample was normalized with the mean and standard deviation of the training set.

To find suitable hyperparameters to train the segmentation model, we conducted a grid search over a range of options for the filter counts and the initial learning rate. The search space was determined experimentally, consisting of seven options for the initial learning rate and three options for the filter counts. For our dataset, the best learning rate was $0.001$, and the best filter counts were (64,128,256,512).

We built a model with the best filter counts and initial learning rate trained it for fifty epochs. Each epoch consisted of $\lfloor 1500/batchsize\rfloor$ training steps, and $\lfloor 500/batchsize\rfloor$ validation steps. At each training step, $batchsize$ samples were chosen at random from the training dataset. At each validation step, $batchsize$ samples were chosen at random from the validation dataset.

To obtain probabilistic segmentations of full resolution images using the model, which takes 320×320 image patches as input, images were divided into overlapping 512×512 patches. Each patch was downsampled to the target size, normalized, and processed by the model, resulting in a probabilistic segmentation of each patch. Subsequently, the overlapping tiled predictions were blended together [25]. The blended prediction value for each overlapping pixel position was computed as the average value of the corresponding pixel in the overlapping tiles.

### Post-Processing Raw Segmentations Prior to Graph Extraction

We post-processed the probabilistic segmentations to remove background noise and enhance vessel centerlines. First, we removed circular regions and small disconnected components from the segmentation. To find these components, we first computed a binary segmentation mask by thresholding the probabilistic segmentation at $p=0.5$, and assigned a component ID to the pixels within each connected component of the mask. We calculated circularity of each component using the formula, $4\pi area \div {perimeter}^{2}$, and removed components whose circularity is greater than $0.8$. Additionally, we computed a 1-pixel wide skeleton [26] of each component of the segmentation mask, and removed components whose skeleton contained no junctions, which indicates that there are no attached branches. After removing these spurious components, we applied the filtered segmentation mask to the probabilistic segmentation to remove background noise. Secondly, we applied another post-processing operation to the probabilistic segmentation to increase the values of the pixels on the expected centerlines of the vessels, relative to the pixel intensities on the edges of the vessels. This step is important because our subsequent processing methods assume the bright ridges on the image represent the centerlines of vessels. This is not the case for the raw probabilistic segmentation, as the class probabilities tend to be uniform along a cross-section of a vessel. To enhance the centerlines of the predicted vessel regions, we computed the medial axis of the segmentation mask and multiplied the segmentation class probabilities by weights computed by a custom distance function. The weight for each pixel was calculated as a function of its Euclidean distance from the nearest point on the medial axis pixel and the Euclidean distance to the nearest background point, per the formula, $\frac{{distance}_{background}}{{distance}_{background}+{distance}_{medial axis}}$. This function effectively computes a distance transform relative to the local vessel width. The probabilistic segmentation was multiplied by these weights to bring vessel centerlines into focus, isolating the appropriate signal for subsequent graph extraction.

### Post-Processing Sato filter Z-Stack Prior to Graph Extraction

We post-processed each Sato-filtered z-stack to obtain a 2D representation while preserving clearly marked vessels at varying depths in the original 3D image. First we applied an unsharp mask to the z-stack to enhance the definition of vessel boundaries at all depths. We then applied max-intensity z-projection to obtain a 2D vesselness filter image.

The following additional steps were performed to produce a binary mask of microvasculature that we later applied to the 2D vesselness filter image as a background elimination technique. To produce this mask, we first applied a Canny edge filter [27] and selected the edge pixels as seed points for region growing. We then applied region growing, using positive gradient as the recursive rule for adding 8-connected neighbor pixels to the mask, to fill in the vessels on the mask, starting from a rough outline (the Canny edges) of the vessels. A morphological closing operation was then applied to fill holes and crevices in the mask. We then labeled connected components on the mask and measured the area and perimeter of each component. We calculated the circularity of each component as a function of area and perimeter using the formula, $\frac{4\pi A}{{P}^{2}}$, and removed components whose circularity is greater than 0.8. Lastly, we applied the binary mask to the vesselness image to isolate prominent vessels for subsequent graph extraction.

### Graph Extraction

To calculate statistics of the endothelial cell microvessel formation, we needed a way of extracting a graph representation (i.e. a list of vertices and edges) from the image. We used the Discrete Persistent Structures Extractor (DisPerSE) algorithm [19] to extract a graph representation of a microvessel network. The DisPerSE algorithm was originally developed as a way of extracting a representation of the “cosmic web’’ of galaxies. It has since been used in Computational Biology as a way of extracting biological networks from image data. For example, it has been used to extract representation of neuronal data from images of neurons. As the DisPerSE algorithm is heavily inspired by the mathematical field of Morse Theory, we refer to the graph representation computed by the DisPerSE algorithm as the *Morse skeleton.*

Intuitively, the DisPerSE algorithm works as follows. We can think of a grayscale image as a “mountain range” in three dimensions, where the x and y coordinates of a pixel are its x and y coordinates in an image, and the z coordinate of a pixel is its brightness. The graph returned by Disperse are the “mountain ridges” connecting different peaks in the mountain range. However, the mountain range of an image may have many more ridges than the network depicted in the image, as noise in the image can add many different peaks of similar height around a “true” peak. Thus, Disperse performs a smoothing step to remove multiple peaks of similar height by “canceling” two nearby peaks and the saddle connecting them.

The key challenge of extracting a graph from our dataset was that the distribution of endothelial cells are non-uniform; rather than forming a line, microvessels are formed and changed over time and based on the material that the endothelial cells are seeded. Therefore, images of microvessels are not of uniform brightness. The DisPerSE algorithm is an appropriate method for extracting a representation of a network from an image as it is well-suited for non-uniform data, as the DisPerSE algorithm can “connect the dots” between bright regions of the image separated by dimmer regions.

However, this ability to “connect the dots” can also be a disadvantage, as DisPerSE will try to connect any bright region to the microvessel network. This can be problematic as images often contain cells which are not a part of the main microvessel network. In this case, DisPerSE will add a path connecting these isolated cells to the main microvessel network. To prevent DisPerSE from returning an overly connected network, we filtered for background noise as described in the previous section.

We then simplified the network extracted by DisPerSE in two ways. First, we removed branches shorter than 10 µm, as these branches are often the result of noise in the original image or segmentation mask. Furthermore, we found that this step was key for returning a network that more closely agreed with human reviewers in terms of number of branches and average microvessel length. Second, we performed moving average smoothing on each of the branches. This is because the graph extracted by the DisPerSE algorithm was a subgraph of the grid connecting neighboring pixels. This means that all of its edges were either vertical, horizontal, or at a 45 degree angle. We found that smoothing the branches gave a graph representation that more accurately traces the underlying microvessel network.

### Topological Data Analysis

**Fig. 4:**
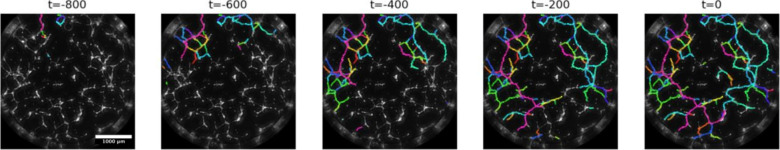
Example of filtration on a Morse skeleton. As the value of t increases, the tree grows from the leaves toward the root. Whenever a new leaf is added, a bar is added to the persistence diagram. When two branches merge, the shorter bar ends in the persistence diagram.

Once we had a graph representation of a microvessel network, our next step was to characterize the branching structure of the microvessels. For example, we wanted to be able to distinguish between networks by the number and lengths of branches. For this task, we needed a way of uniquely decomposing a network into a set of branches (Fig. 4). For this, we relied on the theory of *persistent homology*.

We now present an informal overview of persistent homology. We recommend the book by Edelsbrunner and Harer [12] for a formal introduction to persistent homology. In short, persistent homology is a way of summarizing the topological features of a space across different scales. Generically, persistent homology takes as input a space $X$ and a real-valued function$:X\to R$. It then considers the *filtration* of subspaces ${X}_{t}=\{x\in X : f(x) \le t\}$ as we increase the value $t$. Persistent homology tracks how certain topological properties (such as the number of connected components or loops) changes as we increase the parameter $t$.

The changes in topology are summarized in a *barcode*. A barcode is a collection of intervals $\{[{b}_{i}, {d}_{i}] : 1\le i\le m\}$. Each *bar* $[{b}_{i},{d}_{i}]$ describes the lifetime of a specific topological feature. For example, the feature could be a connected component. The value ${b}_{i}$ is the real number of the space ${X}_{{b}_{i}}$ where the feature first appeared, or was *born*, and the value ${d}_{i}$ is the real number of the space ${X}_{{d}_{i}}$ where the feature merged with an older feature, or *died*. The value $({d}_{i}-{b}_{i})$ is the *persistence* of a feature.

In our example, the space $X$ is the endothelial network, and the value $f(x)$ for a point $x$ in our network is the negative distance from $x$ to a fixed point $r$. It is helpful to visualize the filtration of $f$ in the case that our network is a tree and $r$ is a root. As we increase the value of $t$, we can imagine the space ${X}_{t}$ growing from the leaves towards the root (Fig. 4). Each bar $[{b}_{i}, {d}_{i}]$ corresponds to a branch in a tree. The value ${b}_{i}$ is the negative distance of the leaf from the root, and the value ${d}_{i}$ is the negative distance from the root at the point where the branch merges with a longer branch. The persistence $({d}_{i}-{b}_{i})$ is the length of the branch. This filtration has previously been applied to the task of quantifying the branching structures of neurons [14].

However, it was not always the case that our microvessel network was a tree. In this case, the barcode of the filtration $f$ does not have the same interpretation of decomposing the network into different branches. Specifically, if the network contained a loop, the filtration would not necessarily decompose this loop into two branches. To remedy this, we computed the persistent homology of the shortest path tree from a specified root $r$, where $r$ is chosen to be the center of the network.

We then used the barcode to provide a summary of the branching structure of the network, reporting the number of branches, total length of the network, and average microvessel length (Fig. 3). We compared these metrics with the manual measurements outlined in the section above. focusing on the measurement of average branch length. Specifically, we evaluated the accuracy of the model predictions by calculating the residuals (i.e. Observed Predicted) and the coefficient of determination (R^2^) for the comparison of manual and automated measurements.

### Applying the Software to Naive Imaging Datasets

To demonstrate the broad applicability and utility of the software to microphysiological models in general, the software package was used to calculate cell coverage, microvessel length, and cell invasion for two additional microphysiological models. Image data from the coculture lumen models were kindly provided by the Hind Lab at the University of Colorado Boulder and image data from the coculture microvascular fragment models were kindly provided by the Hettiaratchi Lab at the University of Oregon.

To fabricate the lumen model, immortalized human blood-brain barrier endothelial cells (hCMEC/D3) were purchased from Sigma-Aldrich (St. Louis, MO, USA) and used without additional characterization. Cells were expanded in EndoGRO-MV Complete Culture Media Kit (Sigma) in Type-I collagen (Millipore Sigma, Burlington, MA) coated flasks and were used at passage 5. Human Brain Vascular Pericytes (HBVPs) were purchased from ScienCell ( Carlsbad, CA) and used without additional characterization. Cells were expanded in Pericyte Growth Medium (ScienCell) in Poly-L-Lysine coated flasks and were used at passage 6. Microfluidic devices were fabricated and prepared as previously described [28, 29]. Briefly, polydimethylsiloxane (PDMS) was polymerized over top and bottom silicon wafer masters. The two layers were aligned, a PDMS rod was inserted between them, and the devices were then bonded to a glass bottom dish with oxygen plasma. The microfluidic devices were UV sterilized for 15 min before being incubated with 2% polyethylenimine for 10 min followed by 0.4% glutaraldehyde for 30 min. Type-I collagen was neutralized and diluted to a final concentration of 4 mg/mL and then loaded into the device chamber. After overnight collagen polymerization, the PDMS rod was removed leaving a hollow cylindrical chamber. Before being seeded into the microfluidic device, HBVP cells were incubated in a 1:200 dilution of Vybrant™ DiO in serum free RMPI at a concentration of 106 cells/mL for 20 min in a 37 °C water bath. DiO stained HBVP cells were then seeded into the cylindrical chamber at a concentration of 20,000 cells/µL in PGM. The devices were flipped every 5 min for 30 min before being placed on a rotator overnight. Media was changed the following morning and cells were allowed to culture until 24 h after the initial seeding time. hCMEC/D3 cells were then seeded at a concentration of 20,000 cells/µL in EndoGRO-MV Media and the devices were once again flipped every 5 min for 30 min before being placed on a rotator overnight. EndoGRO-MV Media was changed twice daily for the next 48 h. After 72 total h of culture, HBVP and hCMEC/D3 devices were incubated with pre-warmed 4% paraformaldehyde in PBS for 30 min at room temperature. Paraformaldehyde was removed with three washes of PBS and the devices were then incubated with PBST (PBS with 0.1% Tween 20) for 10 min. After, the devices were stained with Hoescht (1:200, 23491-45-4, Sigma Aldrich, St. Louis, Missouri), Phalloidin (1:1000, ab176757, abcam, Cambridge, UK), and VE-Cadherin (1:120, 130-125-985, Miltenyi Biotec, Bergisch Gladbach, Germany) in PBST overnight at 4 °C. The stained devices were washed with PBS three times the following morning. Single time point confocal Z-stacks were taken with 1 μm steps along the Z-axis with a Nikon A1R HD25 Laser Scanning Confocal Microscope using a Nikon 20x/0.95 (NA) water immersion objective operated by Nikon Elements software.

To fabricate the microvascular fragment coculture model, microvascular fragments (MVFs) were isolated from epididymal fat pads in male Lewis rats as previously described with some minor modifications [30]. All surgical procedures were conducted according to the University of Oregon Institutional Animal Care and Use Committee protocols. The experiment was conducted under the approved protocol “AUP-21-07 - Biomaterials for Microvascular Network Formation.” Briefly, harvested tissues were digested by hand mixing within a 37 °C water bath in a solution containing 5 mg/mL bovine serum albumin (Thermo Fisher, Waltham, MA), 2.3 mg/mL collagenase type 1 (Worthington, Lakewood, NJ) and 1.3 mg/mL DNase I (Roche, Basel, Switzerland). Digested tissue was centrifuged and resuspended in Hank’s Balanced Salt Solution (HBSS; Corning, MA, USA) supplemented with 5% heat-inactivated fetal bovine serum (FBS; R&D Systems, MN, USA). Resuspended MVFs were filtered in sequence through 200 and 20 µm nylon mesh filters to remove single cells and select for fragments between 20 and 200 µm. The nylon mesh was transferred to a sterile petri dish and washed with HBSS-FBS (Cytiva, Chicago, IL) to collect accumulated MVFs. MVFs were seeded into collagen type I hydrogels (0.3% w/v, Corning) at a concentration of 20,000 fragments/mL and cultured in serum-free DMEM + F12 media containing bovine serum albumin (100 µg/mL) transferrin (100 µg/mL), insulin (10 µg/mL), sodium selenite (30 nM), progesterone (20 nM), putrescine (100 µM), and 1% penicillin-streptomycin (Thermo Fisher, Waltham, MA). MVF-seeded gels were incubated at 37 °C and 5% CO_2_, and the media was replaced on days 3 and 5. After 7 days, MVFs-containing collagen hydrogels were fixed with 4% paraformaldehyde and stained with rhodamine-labeled Griffonia simplicifolia lectin I (Vector Laboratories, Newark, CA) before imaging using a Nikon CSU-W SoRa Spinning Disk confocal microscope. Images were analyzed for total network length and branching using Amira Software (Thermo Fisher). Each confocal Z-stack was 3D median filtered and deconvolved before volumetric segmentation and network morphological assessment, as previously described [31].

## Results

### Cell Coverage Area

**Fig. 5:**
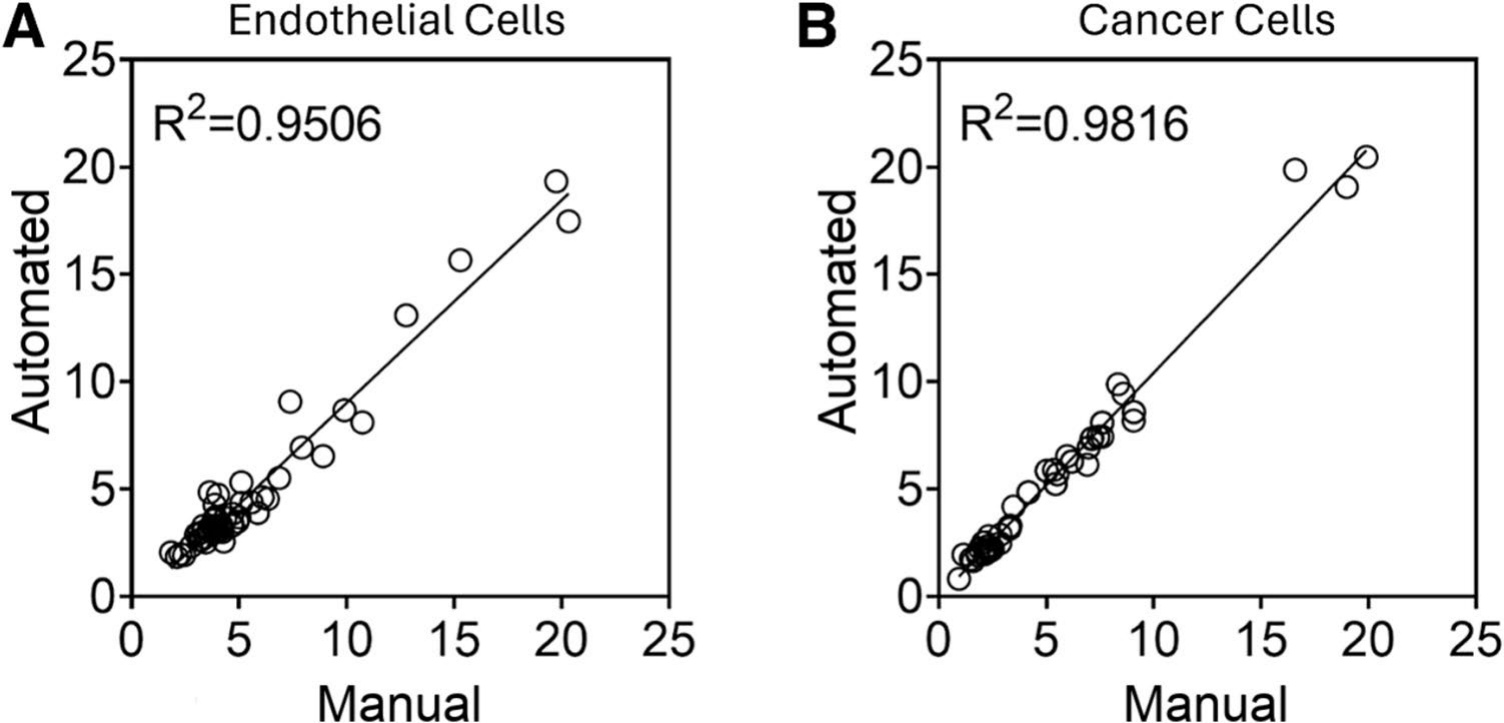
Comparison of Manual measurements and automated pipeline for the percentage of cell coverage. Randomly selected images representing all three coculture models were selected and cell coverage calculated with the automated pipeline was compared to cell coverage calculated manually. This was performed for **A** endothelial cell coverage and **B** cancer cell coverage. N = 58 images.

To evaluate the accuracy of our software application in estimating cell coverage, we compared the computed area measurements with manual area measurements for the multilayer multicellular models of cervical and endometrial cancers. Randomly selected images ranging from the minimum to the maximum of cell coverage representing all three coculture models were used to compare cell coverage area measured manually and with our automated pipeline (Fig. 5). The coefficient of determination (R^2^) was above 90% for both endothelial cells (Fig. 5A) and cancer cells (Fig. 5B).

### Invasion Depth

**Fig. 6:**
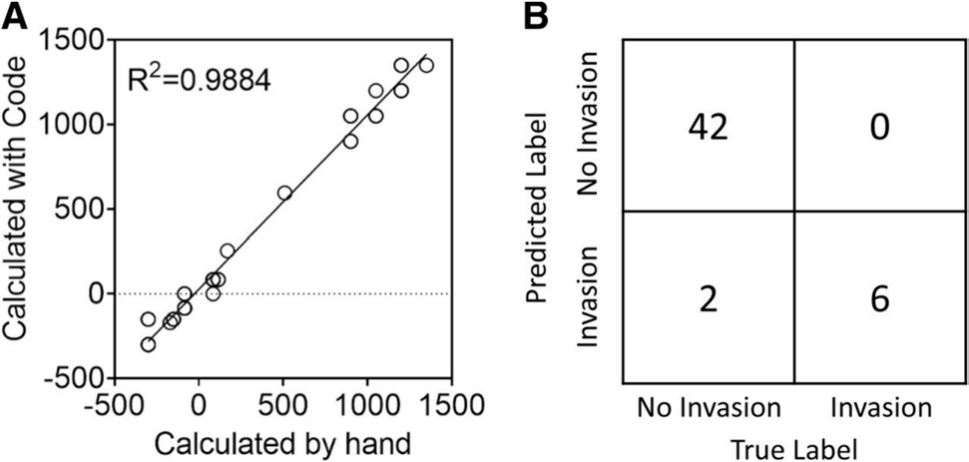
Comparison of cancer cell invasion measured manually and with automated analysis software. Randomly selected images representing all three coculture models were selected to assess the performance of the automated invasion depth pipeline. **A** Comparing the invasion depth determined by the automated pipeline to the invasion depth determined manually. **B** Confusion matrix of cancer cell invasion. N = 50 images.

To assess the accuracy of our software application's invasion depth analysis system, we compared the output of the binary classifier with manual classifications (Fig. 6). Randomly selected images ranging from the minimum to the maximum of cancer invasion representing all three coculture models were used to evaluate the accuracy of invasion depth (Fig. 6A) as well as the accuracy of binary classification whether or not the cancer cells had invaded or now (Fig. 6B). The model had a high prediction on determining the cancer cell invasion of the three cell lines (SiHa, CaSki, and HEC-1A), showing a R^2^ of 0.9884. The binary classification model correctly identified 42 images as not invaded and 6 images as invaded. The model incorrectly identified 2 images as invaded, but it did not misclassify any invaded images. Based on these results, the model demonstrated a classification accuracy of 96%, sensitivity of 100%, and specificity of 95%.

### Microvessel Network

**Fig. 7:**
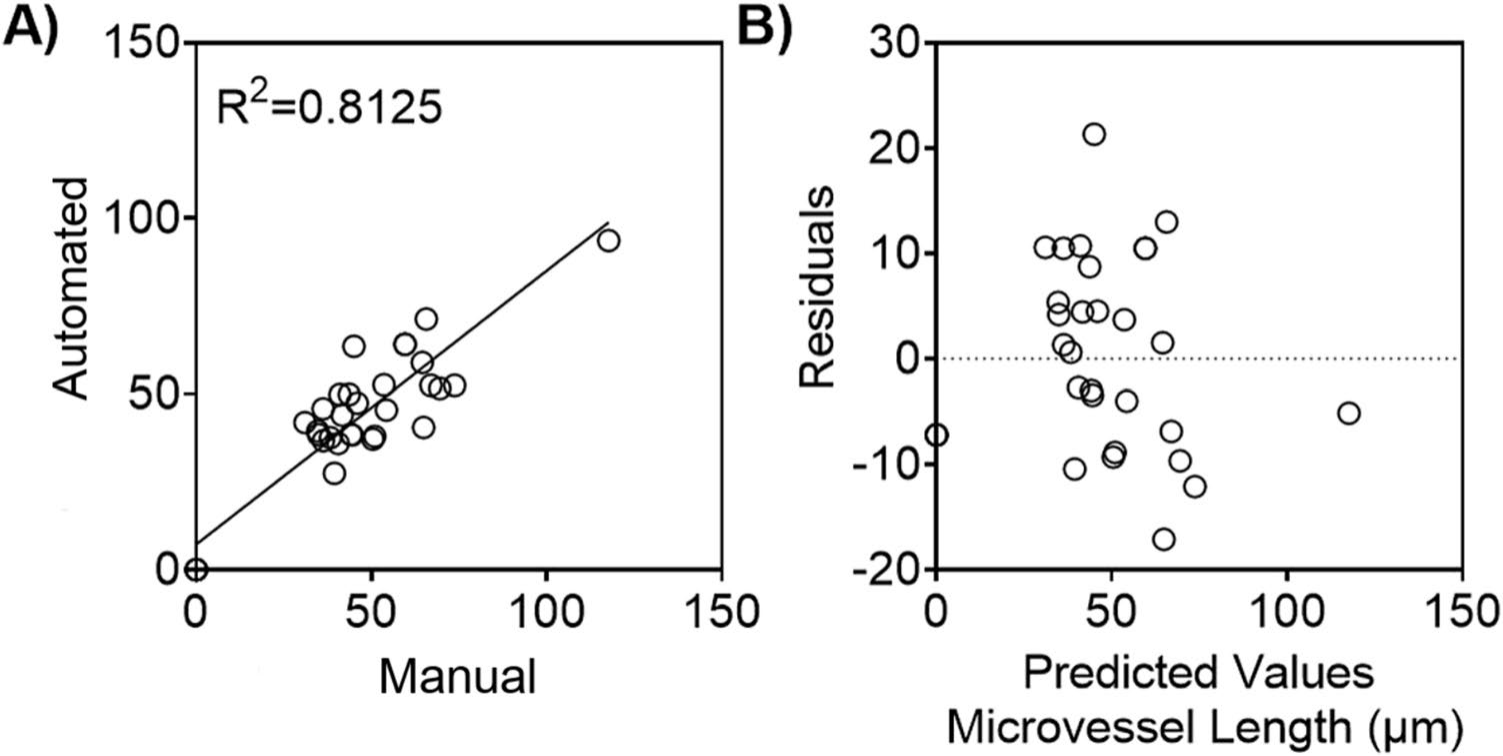
Average microvessel length measured manually and with the automated software. Randomly selected images representing all three coculture models were selected to assess the performance of the automated microvessel analysis pipeline. **A** Comparison of endothelial cell’s microvessel length measured manually using Fiji ImageJ, and algorithm that automatically detects and quantifies the microvessel length** B** Residual plot showing the difference between the ground truth average microvessel length and the metrics computed by the automated pipeline. N = 32 images.

To assess the accuracy of our software application’s microvessel length analysis system, we compared the metrics obtained from the automated branching analysis pipeline with the metrics obtained from manual inspection (Fig. 7). This was done using randomly selected images ranging from the minimum to the maximum of microvessel length representing all three coculture models. The R^2^ value for microvessel length exceeded 80% when comparing measurements predicted by the automated script to those obtained manually in Fiji ImageJ (Fig. 7A). These findings are supported by the results displayed in the residual plot, where the predicted values are dispersed around 0 (Fig. 7B).

### Comparison of Dose-Response Analysis

We performed a comparative analysis of the response of endothelial cells (hMVEC) and the cervical cancer cells (SiHa and Ca Ski) to Paclitaxel. Here we defined IC_50_ as the drug concentration that reduced either cancer invasion, cancer coverage, microvessel length, or endothelial cell coverage to 50% of the value observed in untreated cells. IC_50_ values were derived from the phenotypic metrics measured by our automated image analysis software. These values were then compared to the previously reported values measured manually in Fiji ImageJ and Gen5 [17].

**Fig. 8:**
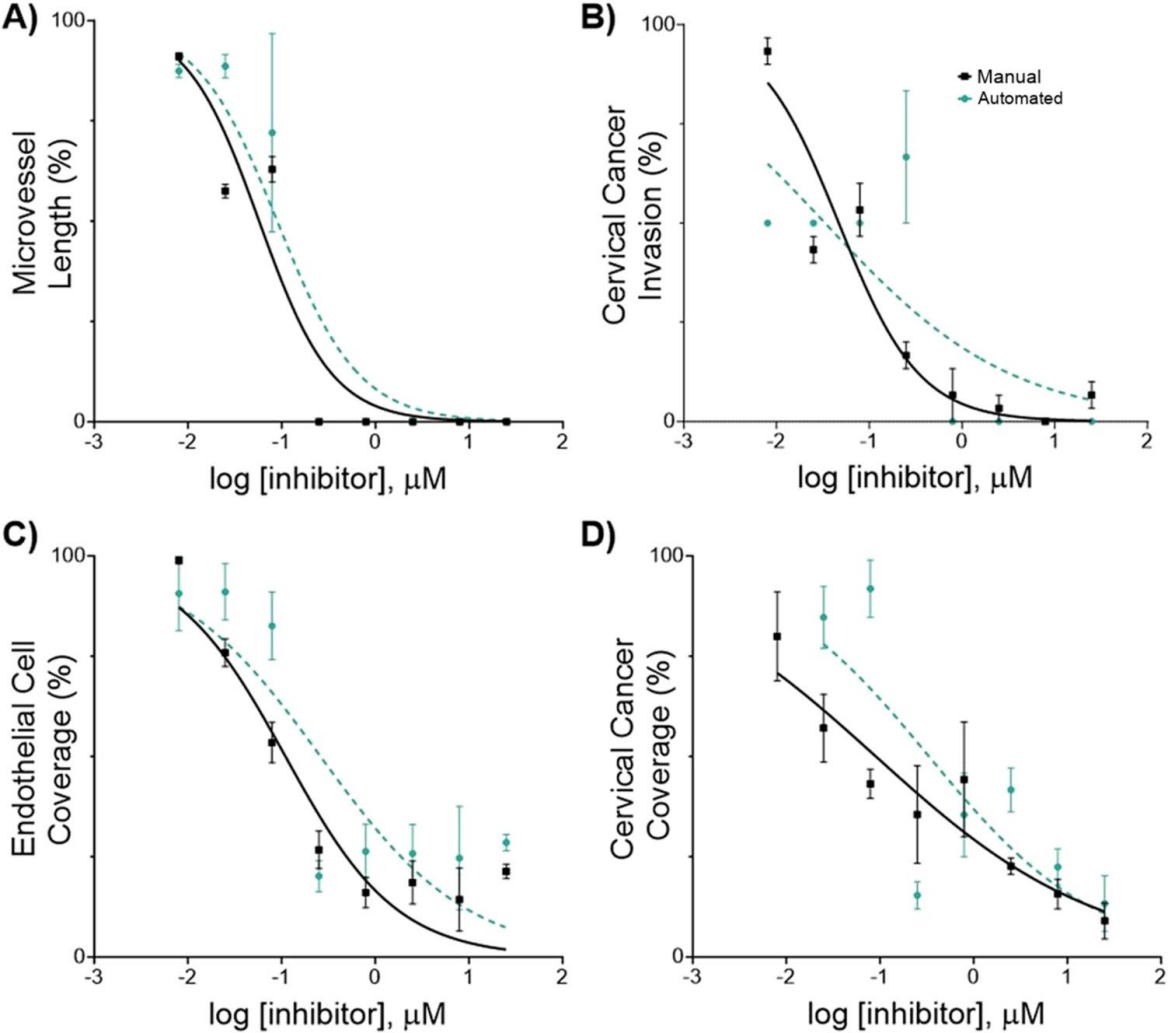
In vitro effects of Paclitaxel on human microvascular endothelial cells (hMVECs) and human cervical cancer cell line (SiHa**).** Phenotypic cell responses were measured using the developed software (automated) and using Fiji ImageJ and the Gen 5 software (manual). Cells were co-cultured in the 3D *in vitro* model for 24 h, then treated with 0.008–25 µM of Paclitaxel for 24 h, at which point cell response was evaluated. Each cell response is normalized to the average of the values observed in the absence of the drug. **A** Microvessel length** B** Cervical cancer invasion **C** Endothelial cell coverage **D** Cervical cancer coverage. Data represent the mean ± SEM (n = 3).

**Fig. 9:**
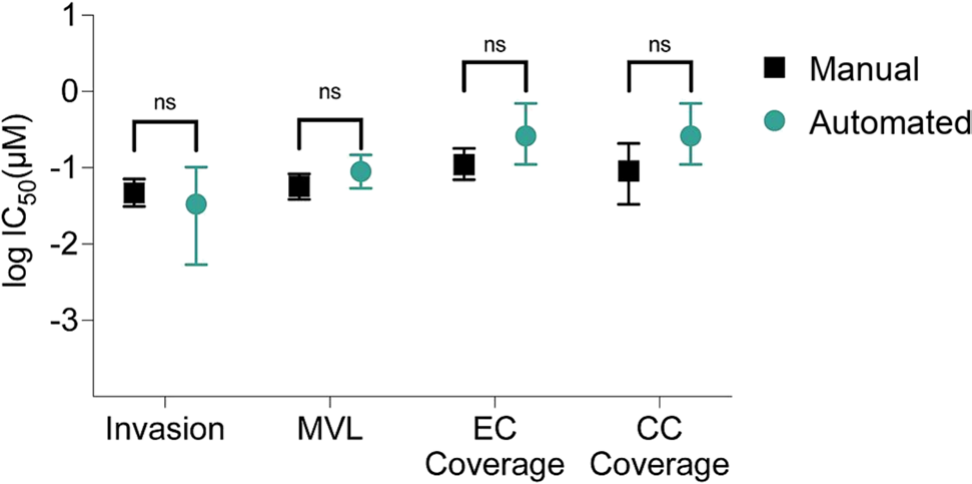
Comparison of drug inhibitory effects (IC_50_) on phenotypic cell responses. Best-fit logIC_50_ values from each inhibitory drug response curve derived from phenotypic response metrics measured manually and with the automated pipeline. Upper and lower limits of the profile likelihood confidence interval on best-fit logIC50 are represented by the error bars. Microvessel length (MVL), EC endothelial cells (EC), cervical cancer cells (CC). Best-fit logIC_50_ values were compared using Welch’s t-test (invasion: p = 0.580, MVL: p = 0.181, EC coverage: p = 0.0786, CC coverage: p = 0.0555).

All cell lines showed a similar dose-response curve compared to the previously reported values measured manually (Fig. 8, Figure S5). Consistent with our prior findings, we reported a higher IC_50_ value for cervical cancer invasion in Ca Ski cells compared to SiHa cells. We compared best-fit log IC_50_ values obtained through manual measurements and our automated image analysis pipeline using Welch's *t*-test, which accounts for the unequal uncertain estimates between best-fit log IC_50_ values (Fig. 9, Figure S6). For SiHa cells, we found no statistically significant differences between best-fit log IC_50_ values derived from manual and automated measurements for any of the four phenotypic responses (invasion: *p = 0.580*, microvessel length: *p = 0.181*, endothelial cell coverage: *p = 0.0786*, cancer cell coverage: *p = 0.0555*). For Ca Ski cells, we only observed a statistically significant difference in the log IC_50_ values derived from endothelial cell coverage measurements (invasion: p = 0.725, microvessel length: *p = 0.260*, endothelial cell coverage: *p = 0.0189*, cancer cell coverage: *p = 0.316*).

### Software Performance on Coculture Lumen and Microvascular Fragment Models

**Fig. 10:**
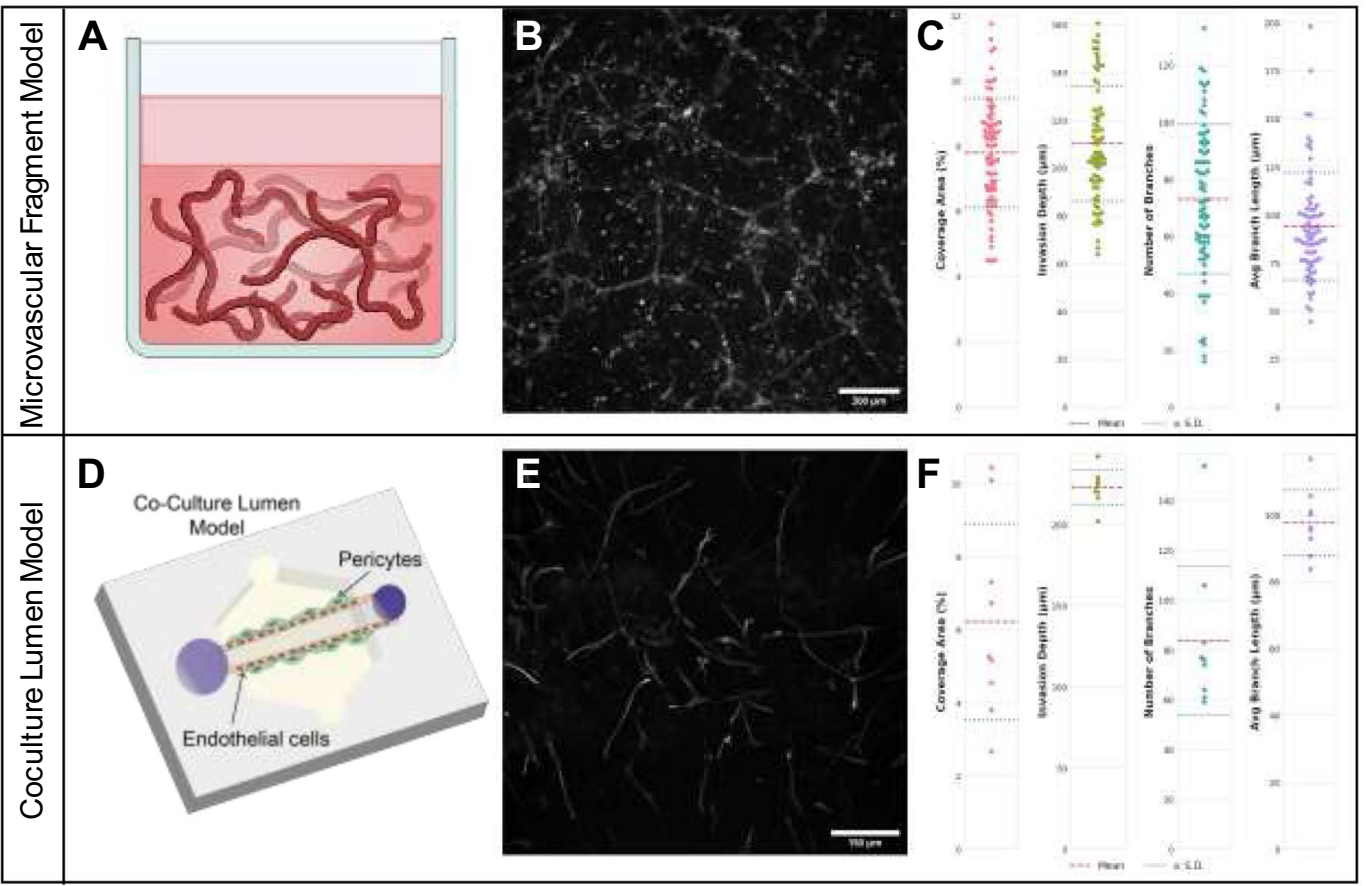
Automated analysis of lumen and microvascular fragment coculture microphysiological models**. A** Schematic of microvascular fragments in a Col1 hydrogel. **B** Representative maximum intensity projection of a z-stack image from a microvascular fragment model. Scale bar represents 200 μm. **C** Quantitative measurements of cell coverage area, invasion depth, number of microvessel branches, and average microvessel branch length from the microvascular fragment model dataset (n = 72 z-stacks). **D** Schematic of coculture lumen model with pericytes and endothelial cells in a Col1 hydrogel. **E** Representative maximum intensity projection of a Z-stack image from a coculture lumen model showing hCMEC/d3 cells and human brain vascular pericytes stained for actin filaments. Scale bar represents 150 μm. **F** Quantitative measurements of coverage area, invasion depth, number of microvessel branches, and average microvessel branch length for the coculture lumen dataset (n = 9 Z-stacks). In B and D, each dot represents a measurement from a single Z-stack, red dashed lines indicate mean values, and blue dotted lines show ± standard deviation.

To ensure that the software was broadly applicable beyond the original multilayer multicellular platforms, we evaluated confocal images from two additional microphysiological models, a microvascular fragment model and a coculture lumen model, both in a 3D collagen network. The software was able to calculate cell coverage, invasion depth, number of endothelial branches, and average endothelial branch length for both the microvascular fraction model (Fig. 10 A–C) and the coculture lumen model (Fig. 10 C–E).

## Discussion

In this work, we developed an open-source Python software application designed for the automated analysis of dynamic cell behaviors in hydrogels. We integrate computer vision, machine learning, and topological data analysis to efficiently quantify cell coverage area, invasion depth, and microvessel network characteristics. This software automatically generates measurements that correlate highly to manual measurements, but with significantly reduced analysis time.

As a complement to popular open source bioimage analysis tools such as Fiji ImageJ, our software is targeted to address the distinct challenges in *high throughput* measurement of these complex and meaningful phenotypic parameters. We designed our automated analysis pipeline as a general tool that can optionally be configured and trained, yet produces meaningful output on unseen images using the default configuration and pretrained model. This low-configuration approach differs from typical automated workflows and ML applications in image analysis [5, 6]. Through extensive data augmentation, we trained and validated our ML models on fewer than 1000 binary annotations for invasion depth and 50 binary masks for microvessel segmentation.

Automated tools can facilitate consistent and standardized assessments of phenotypic characteristics, and are not prone to cognitive biases such as inattentional blindness [32]. However, it is a good idea to manually inspect the pipeline’s intermediate outputs and image transformations to evaluate its assessments and tune its configuration parameters. Our software facilitates visual evaluation of its assessments by saving intermediate output visualizations for later review.

To validate our automated measurements, we needed to approximate the ground truth measurements based on the image data. The manual techniques we chose involve steps that can introduce errors and bias, such as visual identification of image features, manual determination of image thresholding parameters, and by-hand region annotation. Unfortunately, all image data analysis approaches used to infer real-world properties are limited by the information represented in the image data, which might present a noisy or complex signal requiring a subject matter expert to interpret. For instance, a large portion of our images analyzed in section "Comparison of Dose-Response Analysis" contained only a small number of short vessels interspersed with cell clumps, which are difficult to tell apart. This scenario presented a challenge for both automated and subject matter expert-performed manual measurement of microvessels, leading to increased variability in the measurements for branch count and lengths. We believe that this issue explains a large portion of the variability between manual and automated measurements of microvessel counts and length in our comparison of dose-response analysis (Figure S4).

We extract a 2D embedded graph from images to analyze microvessels. This procedure preserves vessels at variable depth, but it does not fully preserve paths of overlapping vessel segments. In cases where a vessel branch is routed under or over a different vessel branch, only the most prominent branch is retained. This technique was designed to be efficient, but it is less effective on Z-stacks containing overlapping or steeply angled microvessels. Future work could perform end-to-end 3D analysis to avoid compressing vessel information to 2D, albeit at a potentially higher processing and memory cost.

Our image analysis tools report basic metrics relating to cell area, invasion depth, and vessel formation. These are useful metrics, but they only represent a small subset of meaningful phenotypic parameters that could be extracted. For instance, our software uses persistent homology to compute a decomposition of endothelial networks into a set of branches. This allows researchers to assess the branching structure of the network. However, in addition to decomposing a network into branches, persistence barcodes have a rich mathematical structure with well-defined distances [33, 34] and kernels [35] between two barcodes. These properties give us ways of comparing pairs of persistence diagrams; for example, we could use these tools to compare the branching structure of endothelial networks over time or before/after treatment. Additionally, these properties mean that ML algorithms like $k$-medoids clustering and support-vector machines can be applied to barcodes. This could enable future work to perform different machine learning tasks using the barcodes output by our software.

Lastly, we only consider a single filtration of the endothelial networks. It has been demonstrated [36] that other filtrations provide useful information of networks beyond their branching properties, e.g. their tortuosity. Future work could consider other filtrations of the endothelial networks to extract these properties.

In conclusion, this work introduces an open-source package designed to automate the analysis of 3D tumor models. Our software quantifies cell coverage, invasion depth, and microvessel formation by integrating machine learning and topological data analysis techniques. Future work could build on our automated framework to extract different phenotypic parameters and to improve adaptability to different imaging conditions. We encourage public contributions to the software application on GitHub to improve its capabilities: https://github.com/fogg-lab/tissue-model-analysis-tools/tree/main/

## Supplementary Information

Below is the link to the electronic supplementary material.

Supplementary file1 (DOCX 749 kb)

## Data Availability

All of the images, data, and code along with comprehensive documentation detailing setup and usage instructions for the image analysis software can be found in our GitHub repository: https://github.com/fogg-lab/tissue-model-analysis-tools.
