# Supplementary material for: Empowering High-Throughput High-Content Analysis of Microphysiological Models: Open-Source Software for Automated Image Analysis of Microvessel Formation and Cell Invasion
Source: Cell Mol Bioeng. 2024 Oct 10;17(5):369–83. doi: 10.1007/s12195-024-00821-2 (PMC11538109; doi:10.1007/s12195-024-00821-2)
Supplement: Supplementary file 1 — Supplementary file1 (DOCX 749 kb) [file 12195_2024_821_MOESM1_ESM.docx]

## **Supplemental Figures**


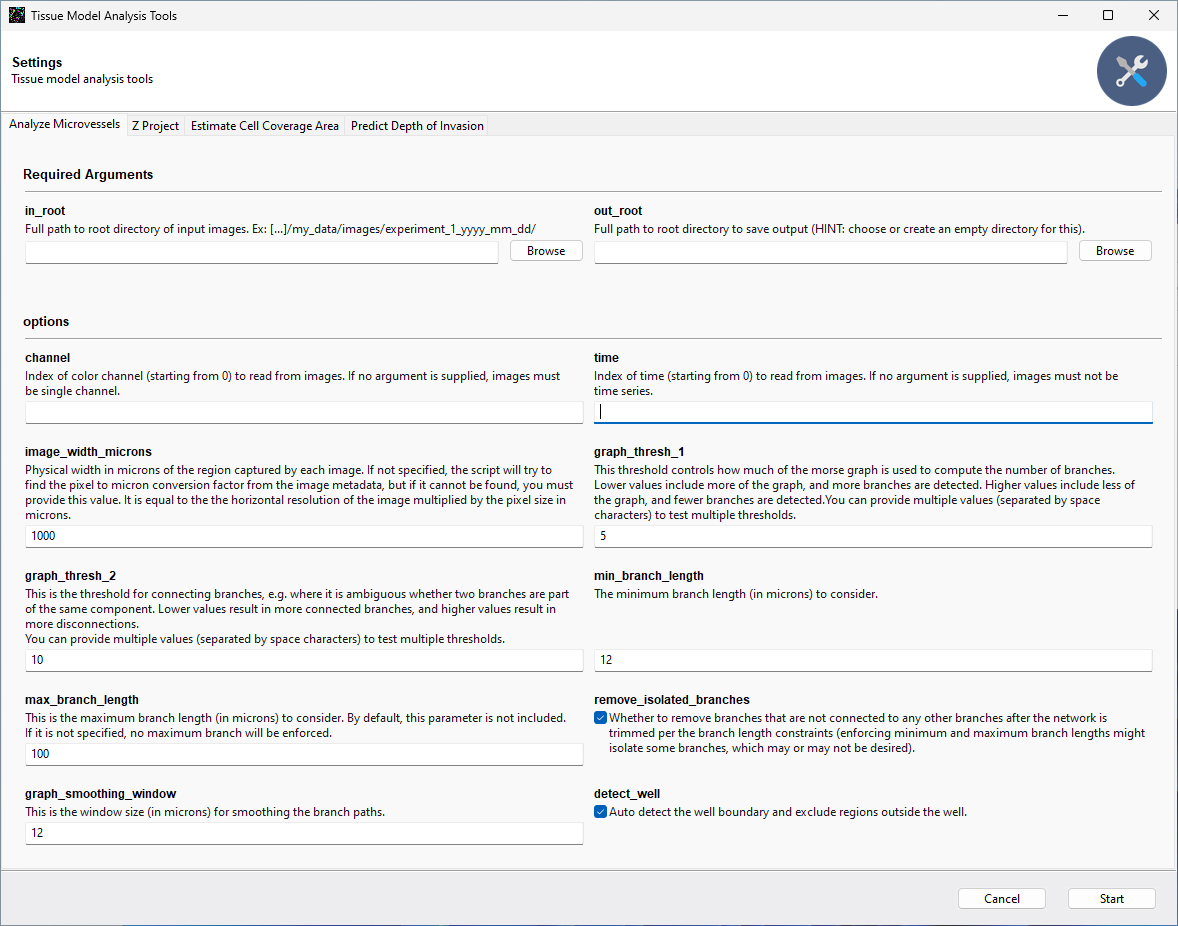


**Figure S1. Graphical user interface (GUI) for the automated image analysis software**

| 1  2  3  4  5  6  7  8  9  10  11  12  13  14  15  16  17  18  19  20  21  22  23  24  25  26  27  28  29  30  31  32  33  34  35  36  37  38  39 | *// Helper ImageJ macro for manual measurement of cell coverage area*  for (w = 0; w < 4; w++) {  run("8-bit");  setAutoThreshold("Li dark");  call("ij.plugin.frame.ThresholdAdjuster.setMode", "B&W");  waitForUser("Create a circle...");  roiManager("Add");  for (p = 0; p < 4; p++) {  waitForUser("Set threshold...");  *// sequence used to find the area covered*  run("Create Selection");  roiManager("Add");  roiManager("Select", 0);  roiManager("Select", newArray(0,1));  roiManager("AND");  roiManager("Add");  roiManager("Select", 0);  run("Measure");  roiManager("Select", 2);  run("Measure");  waitForUser("Waiting for user to copy and paste data...");  *// clear the measurements*  Table.deleteRows(0,1);  roiManager("Select", newArray(1,2));  roiManager("Delete");  *// deselect any current selections*  run("Select None");  run("Save");  run("Open Next");  run("8-bit");  *// prepare for the next iteration*  roiManager("Select", 0);  setAutoThreshold("Li dark");  call("ij.plugin.frame.ThresholdAdjuster.setMode", "B&W");  }  roiManager("Deselect");  *// delete old circle from the ROI so a new one can be made*  roiManager("Delete");  } |
| --- | --- |

**Figure S2. Manual inspection of cell coverage area**. In NIH Fiji-ImageJ, z-projected images were processed using the ImageJ macro script to determine endothelial cell coverage and cancer cell coverage over time.

| 1  2  3  4  5  6  7  8  9  10  11  12  13  14  15  16  17  18  19  20  21  22  23  24  25  26  27  28  29  30  31  32  33  34  35  36  37  38  39  40  41  42  43  44 | *// Helper ImageJ macro for manual measurement of microvessel lengths.*  *// Potential set up for the auto line listener (left commented out)*  *// macro "LL" {}*  for (p = 0; p < 90; p++) {  *// p is the number of photos that need checking*  *// macro "mathtool" {}*  if (getBoolean("Are there any tubes?")) {  Name = getTitle();  roiManager("Show All with labels");  *// Find the tubes. Use control+T to add each line to the ROI*  waitForUser("Measure all the tubes, then click OK.");    count = roiManager("count"); *// Counts the tubes found*  print("File: "+ Name);  print("Number of Tubes: "+ count);    *// grab the points since ROI acts weird with measure all*  numberA = Array.getSequence(count);  *// Array.print(numberA); uncomment for debugging*  roiManager("Select", numberA);  *// selects all the ROI lines and measures them*  roiManager("Measure");    L = Table.getColumn("Length");  print("Tube data list that can be checked for accuracy:");  Array.print(L);  Array.getStatistics(L, min, max, mean, stdDev);    print("The average of these lines are: "+ mean);    waitForUser("Copy and paste the data from the Log");    *// clearing measurement data*  *// there should only be (data found + 4) rows*  Table.deleteRows(0, count);  roiManager("Deselect");  roiManager("delete");  run("Select None");  run("Open Next");  } else {  run("Open Next");  }  } |
| --- | --- |

**Figure S3. Manual quantification of microvessel length**. In Fiji ImageJ, z-projected images were processed using the ImageJ macro script to determine the count and lengths of microvessels over time.


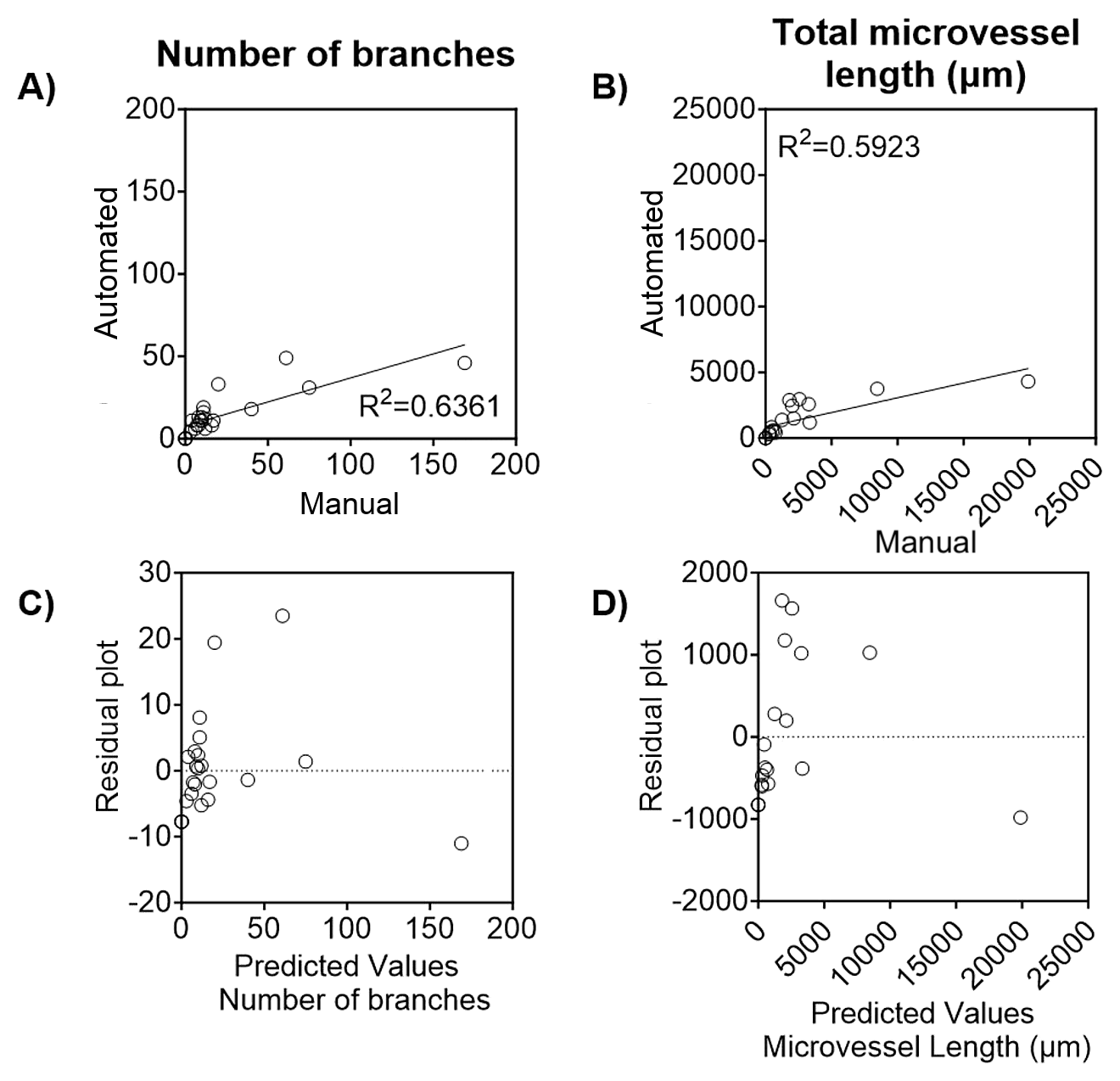
**Figure S4. Average number of branches and total microvessel length measured manually and with the automated image analysis software.** Comparison of endothelial cells’ number of branches and total microvessel length measured manually using Fiji ImageJ, and by our automated pipeline **(A, B)**. Residual plot shows the differences between the manual and automated measurements for (**C**) number of branches, and (**D**) total microvessel length. N=32 images were randomly selected from the 3D *in vitro* models of cervical cancer and endometrial cancer.


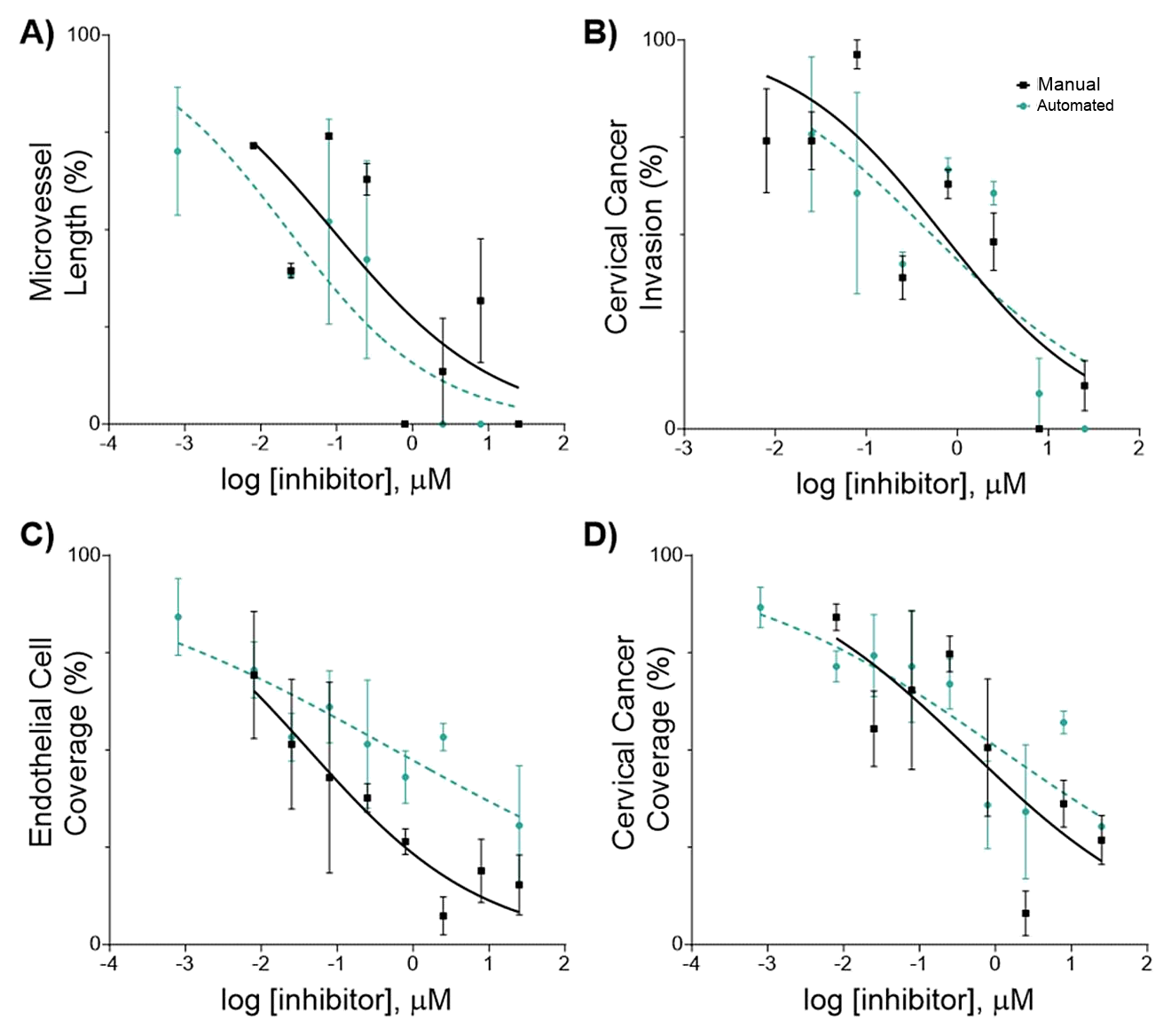


**Figure S5.** *In vitro* growth-inhibitory effects of the inhibitory drugs on human microvascular endothelial cells (hMVECs) and human cervical cancer cell line (CaSki). Phenotypic cell response measured manually in Fiji and with the automated pipeline. Cells were seeded in the construct, cultured for 24 hours, then treated with 0.008 – 25 µM of Paclitaxel for 24 hours, at which point cell response was evaluated. Each cell response is normalized to the average of the values observed in the absence of the drug. **(A)** Microvessel length **(B)** Cervical cancer invasion **(C)** Endothelial cell coverage **(D)** Cervical cancer coverage. Data represent the mean ± SEM (n = 3).


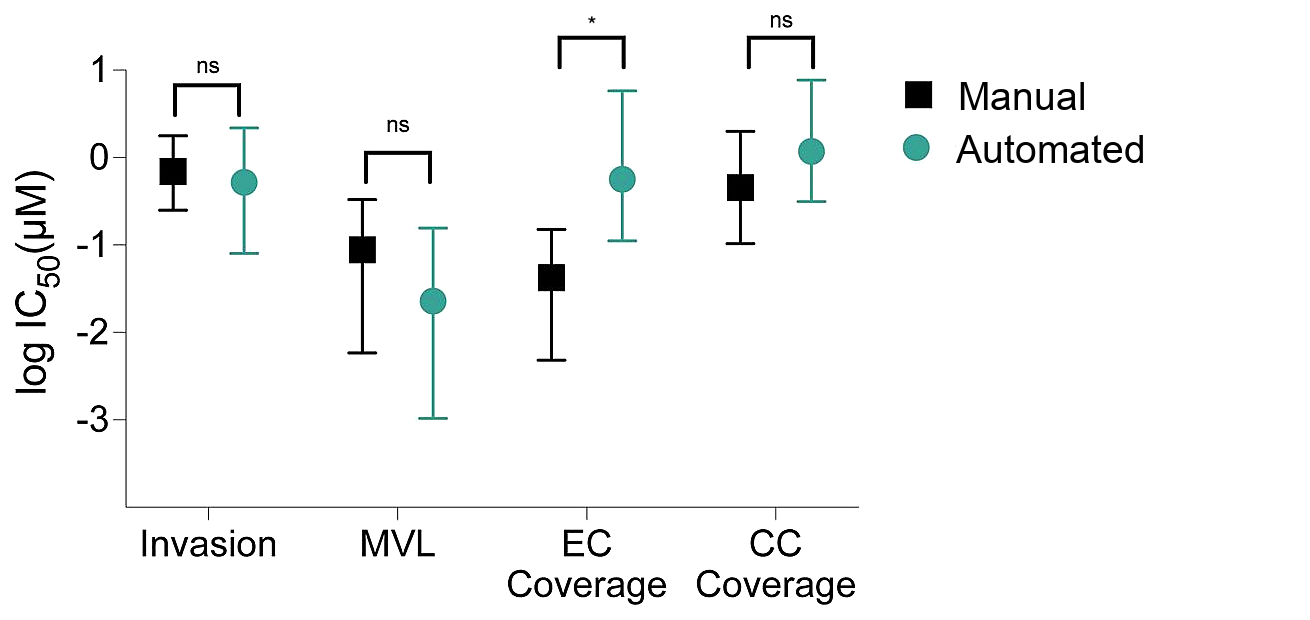


**Figure S6. Comparison of drug inhibitory effects (IC_50_) on phenotypic cell responses.** Best-fit logIC_50_ values from each inhibitory drug response curve derived from phenotypic response metrics measured manually in Fiji and with the automated pipeline. Upper and lower limits of the profile likelihood confidence interval on best-fit logIC_50_ are represented by the error bars. Microvessel length (MVL), endothelial cells (EC), cervical cancer cells (CC). Best-fit logIC_50_ values were compared using Welch’s t-test (invasion: p = 0.725, MVL: p = 0.260, EC coverage: p = 0.0189, CC coverage: p = 0.316).
